# Breaching the Delivery Barrier: Chemical and Physical Airway Epithelium Disruption Strategies for Enhancing Lentiviral-Mediated Gene Therapy

**DOI:** 10.3389/fphar.2021.669635

**Published:** 2021-04-26

**Authors:** Alexandra McCarron, Nigel Farrow, Patricia Cmielewski, Emma Knight, Martin Donnelley, David Parsons

**Affiliations:** ^1^ Adelaide Medical School, University of Adelaide, Adelaide, SA, Australia; ^2^ Robinson Research Institute, University of Adelaide, Adelaide, SA, Australia; ^3^ Department of Respiratory and Sleep Medicine, Women's and Children's Hospital, Adelaide, SA, Australia; ^4^ School of Public Health, University of Adelaide, Adelaide, SA, Australia; ^5^ South Australian Health and Medical Research Institute, Adelaide, SA, Australia

**Keywords:** lentiviral vector, gene therapy, airway, epithelium, rat

## Abstract

The lungs have evolved complex physical, biological and immunological defences to prevent foreign material from entering the airway epithelial cells. These mechanisms can also affect both viral and non-viral gene transfer agents, and significantly diminish the effectiveness of airway gene-addition therapies. One strategy to overcome the physical barrier properties of the airway is to transiently disturb the integrity of the epithelium prior to delivery of the gene transfer vector. In this study, chemical (lysophosphatidylcholine, LPC) and physical epithelium disruption using wire abrasion were compared for their ability to improve airway-based lentiviral (LV) vector mediated transduction and reporter gene expression in rats. When *luciferase* expression was assessed at 1-week post LV delivery, LPC airway conditioning significantly enhanced gene expression levels in rat lungs, while a long-term assessment in a separate cohort of rats at 12 months revealed that LPC conditioning did not improve gene expression longevity. In rats receiving physical perturbation to the trachea prior to gene delivery, significantly higher *LacZ* gene expression levels were found when compared to LPC-conditioned or LV-only control rats when evaluated 1-week post gene transfer. This proof-of-principle study has shown that airway epithelial disruption strategies based on physical perturbation substantially enhanced LV-mediated airway gene transfer in the trachea.

## Introduction

Airway gene-addition therapy is currently under development for treatment of a range of hereditary and acquired pulmonary disorders. Of particular interest is the development of a gene therapy to treat airway disease caused by the genetic disorder cystic fibrosis. Gene-addition therapy employs a vector (non-viral or viral) to deliver wild-type gene copies to the relevant airway cells, with the ultimate goal of correcting the disease pathophysiology that results from the defective gene. While gene vectors can be readily delivered into the lung airways, physical, anatomical, and immune barriers have evolved to protect the host airway cells against airborne pathogens. These natural defences are also directed towards gene transfer vectors, thus limiting gene transduction in the airway epithelium.

Major hurdles to efficient viral-vector mediated gene transfer include a polarised epithelium, paucity of viral receptors on the apical membrane, and the presence of airway tight-junctions that prevent vectors from accessing receptors located on the basolateral side (Copreni et al., 2004). One way to overcome these barriers is to perform epithelial perturbation prior to, or in conjunction with gene transfer vector delivery. Perturbation methods act to disrupt tight-junction integrity, allowing vector particles access to basolateral receptors, thus enhancing vector-mediated gene transfer (Johnson et al., 2000; Pickles, 2004; Kim et al., 2016). Epithelial perturbation can also potentially expose cells for transduction that are not in direct contact with the airway lumen, particularly the basal cells, which function as tissue-specific stem cells (Rock et al., 2009).

To achieve basolateral access, airway conditioning with a chemical “tight-junction opener” can be employed to increase paracellular permeability. Airway conditioning with compounds including ethylene glycol tetraacetic acid (EGTA) (Chu et al., 2001), perfluorochemicals (Li et al., 2007), sodium caprate (Gregory et al., 2003), and sulphur dioxide inhalation (Johnson et al., 2000) have proven effective for disrupting tight-junctions and increasing viral-vector mediated gene transfer in proof-of-principle animal studies (Castellani and Conese, 2010). Another compound that has been investigated extensively for this purpose is lysophosphatidylcholine (LPC) (Limberis et al., 2002; Koehler et al., 2005; Cmielewski et al., 2010; Cao et al., 2013; Cmielewski et al., 2014). LPC is a natural component of lung surfactant that can be used to transiently open cellular tight-junctions when applied to the airway surface. A two-step dosing regimen employing LPC conditioning prior to the delivery of a HIV-1-derived lentiviral (LV) vector pseudotyped with the vesicular stomatitis virus glycoprotein (VSV-G) has proven effective at increasing airway gene transfer in mouse nasal airways (Cmielewski et al., 2010; Cmielewski et al., 2014), but has not yet been assessed in rat lower airways.

Chemical conditioning approaches have disadvantages when it comes to performing LV-mediated gene transfer. LV vectors are highly fragile due to the presence of an outer lipid envelope (Bandeira et al., 2012) and can be inactivated upon contact with conditioning agents such as LPC. Although two separate administrations–one to deliver the conditioning compound, and then a second to deliver the vector after the chemical effect occurs–can be used to enable LV vectors to take advantage of this delivery enhancement effect, this two-step process increases procedure time and complexity. Additionally, transduction may be variable as there is no guarantee that the conditioning compound and the vector will localise to the same regions (Flotte et al., 2007).

An alternative strategy to disrupt the integrity of the airway epithelium is *via* physical perturbation techniques. An early airway gene therapy study employing an adenoviral vector demonstrated that externally applied mechanical injury to the airway epithelium *via* use of fine forceps is a simple and effective way to produce strong reporter gene expression in the trachea of mice. Mechanical perturbation also resulted in high levels of basal cell transduction as this technique removed the superficial transduction-resistant columnar cells thus exposing underlying basal cells to the vector (Pickles et al., 1996). While early studies validated the potential efficacy of a physical perturbation strategy, it has not been investigated further for pre-clinical development, likely due to the perceived impracticalities of performing controlled airway-surface perturbation in animal models. To our knowledge there are no reports of physical airway injury being used in combination with LV vectors for *in vivo* gene delivery.

In this study, physical airway epithelium perturbation was explored as a strategy to improve *in vivo* gene transfer levels in the trachea of rats and this method was compared to standard LPC-conditioning and a perturbation-free LV-only control group. Additionally, we provide the first evidence of the effectiveness of LPC conditioning and LV vector delivery in rat lungs over both short-term (1-week) and long-term (12-month) durations.

## Materials and Methods

Multiple treatment groups were employed in this project. The first study assessed whether LPC had an effect on short- and long-term gene expression levels. In this study, rats received LPC conditioning or a PBS-sham treatment, followed by a HIV-1 derived EF1α-3XFLAG-fLuc-F2A-eGFP vector (LV-FLAG-Luc-GFP) vector. In one cohort (n = 6 per group) *luciferase* expression was measured 1-week following gene delivery, and in a separate cohort (n = 12 per group) expression was measured 12-months after LV delivery. To investigate and compare airway epithelial disruption strategies, separate groups of rats received either no epithelial disruption (control group), physical perturbation, or standard 0.1% LPC conditioning, followed by delivery of a HIV-1-MPSV-nlsLacZ vector (LV-LacZ) (n = 6 per group). Gene expression levels were assessed 1-week post gene transfer in this cohort of animals, as it was expected that the damaged airway epithelium would be largely regenerated and that gene expression would be readily apparent by this stage (Shimizu et al., 1994; Pickles et al., 1996).

### Lentiviral Vector Production

LV vector was produced using previously described methods (Rout-Pitt et al., 2018; McCarron et al., 2019). The bicistronic LV-FLAG-Luc-GFP vector expressed firefly *luciferase* and enhanced *green fluorescent protein* (eGFP) genes under control of the EF1α promoter, together with three epitope FLAG tags. The LV-LacZ vector construct expressed a nuclear-localised β-*galactosidase* gene directed by the MPSV (myeloproliferative sarcoma virus) promoter. LV-FLAG-Luc-GFP was titered by quantifying the proportion of GFP positive cells using flow cytometry (McCarron et al., 2019). The titre of the LV-LacZ vector was determined using real-time quantitative PCR as previously described (Anson et al., 2007).

### Animals

All animal procedures were approved by the University of Adelaide Animal Ethics Committee under applications M-2016–086 and M-2019–061. Female and male Sprague Dawley rats aged >8 weeks were used. For all LV-dosing procedures, rats were anaesthetised with medetomidine (0.4 mg/kg) and ketamine (60 mg/kg) by intraperitoneal injection. Following anaesthesia, the rats were non-surgically intubated with a 16-gauge intravenous cannula (Terumo, SR-FF1651, Japan) acting as an endotracheal (ET) tube, positioned with the tip just past the vocal cords so that the majority of the tracheal tissue was exposed to the conditioning or perturbation and LVvector. Rats that received physical airway perturbation were given subcutaneous administration of meloxicam (1 mg/kg) for precautionary pain relief. After procedure completion anaesthesia was reversed with atipamezole (1 mg/kg).

### Intratracheal Administration of LPC and Lentiviral Vector

Animals were placed in a supine position and received either 25 µL of 0.1% LPC (Sigma-Aldrich, L4129, USA) or PBS. Fluid was administered to the trachea *via* the ET tube using a gel-loading pipette tip that was lengthened with fine polyethylene tubing marked to reach the distal tip of the ET tube. LV vector delivery was performed either without any pre-treatment or one hour following LPC conditioning (or PBS-sham) as this is the time when LPC epithelial tight-junction opening effects are apparent (Cmielewski et al., 2010). LV-FLAG-Luc-GFP (1 × 10^9^ TU/mL) or LV-LacZ (2 × 10^9^ TU/mL) were delivered to the trachea in two aliquots of 25 µL (50 µL total per animal).

### Physical Epithelial Perturbation

**FIGURE 1:**
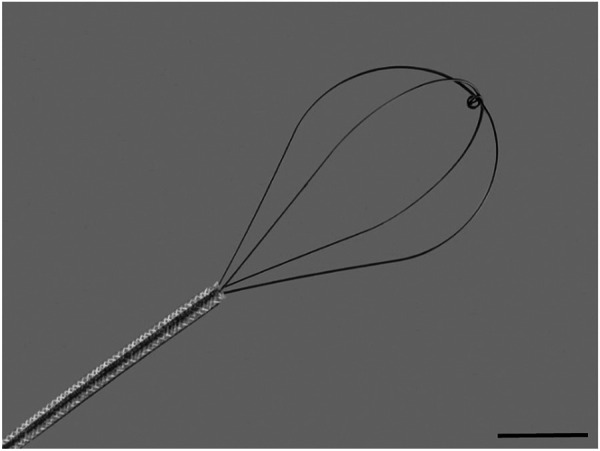
Flexible wire basket used to physically perturb the tracheal airway epithelium. The basket is 1 cm in diameter at full expansion and 1.5 cm in length. Scale bar = 5 mm.

With the intubated animal in the supine position, a pliable wire-basket (NCircle® Nitinol Tipless Stone Extractor NTSE-022115-UDH, Cook Medical, USA; Figure 1) was fed in the retracted position down the ET tube to approximately the carina (based on an average trachea length measurement). The basket was then extended, fully expanded, and drawn up and down the trachea (approximately 10 times per animal) for 30 seconds followed by withdrawal. Two 25 µL aliquots of LV-LacZ (2 × 10^9^ TU/mL) were delivered *via* the ET tube within ten minutes of the perturbation event.

### Bioluminescence Imaging (BLI)

Either 1-week or 12-months following LV-FLAG-Luc-GFP delivery rats were anaesthetised with 2.5% inhaled isoflurane. Once unconscious, 200 µL of 15 mg/mL D-luciferin (Cayman Chemicals, United States) was delivered to the nostrils as a bolus. Each animal was imaged in the supine position (IVIS Lumina XRMS *in vivo* imaging system, PerkinElmer, United States) while maintained with 1.5–2% isoflurane. Animals were imaged with an automatic exposure at 5-minute intervals for up to 15 minutes post D-luciferin delivery. The animals were then humanely killed with intraperitoneal delivery of sodium pentobarbital (150–300 mg/kg). The trachea and lungs were immediately excised and imaged separately in a petri dish containing PBS. Bioluminescence flux (photons/second) was measured using the contour tool (Living Image, version 4.7.2, PerkinElmer, United States).

### Assessment of LacZ Gene Expression

Animals were humanely killed by carbon dioxide asphyxiation and the airway tissues harvested for LacZ staining. Tissues were fixed in 4% PFA and stained for β-galactosidase (LacZ) activity using X-gal as previously described (Cmielewski et al., 2017). Following X-gal staining, the trachea was cut and opened longitudinally, and separated into two halves. *En face* images of the tissue were acquired with a Nikon SMZ1500 stereo microscope; DigiLite 3.0 MP Camera and TC capture software (Tucsen Photonics, China). Illumination intensity was not altered between samples. Tracheas were subsequently paraffin-embedded, sectioned at 5 μM, and counterstained with nuclear fast red. Histology images were captured with a Nikon Eclipse E400 microscope, DS-Fi2-U3 camera and NIS-elements D software (version 5.20.00).

### Immunohistochemistry

X-gal stained sections were deparaffinised and subjected to heat-mediated antigen retrieval in sodium citrate buffer (10 mM sodium citrate, 0.05% Tween 20, pH 6.0) at 95–100°C for 20 minutes. A peroxidase blocking step was performed using 3% hydrogen peroxide followed by permeabilisation with 0.3% Triton X-100 for 10 minutes. Protein blocking was performed with 10% goat serum in PBS. Primary and secondary antibodies were diluted in 1% bovine serum albumin and 0.1% Triton X-100 in PBS. Sections were probed with rabbit anti-cytokeratin 5 antibody (Abcam, ab52635, UK) (1:400 dilution) and were incubated overnight at 4°C. Tissues were washed and subsequently incubated with goat anti-rabbit IgG HRP (Abcam, ab97051, UK) (1:500 dilution) for 1 hour at room temperature followed by staining with 3,3′-Diaminobenzidine.

### Digital Quantification of LacZ Staining in en Face Trachea Images

The amount of LacZ staining in the *en face* images of the trachea was quantified using a custom-written script in Matlab (R2020a, MathWorks) that calculated the area of the trachea that was the specific shade of blue/green of LacZ-positive cells. Briefly, images were converted from the RGB to HSV colour space, and separate thresholds were applied to the hue (0.45 < H < 0.6) and saturation (s > 0.4) channels. The value channel was not used because the illumination across the sample was uneven and did not help discriminate the transduced cells. A binary mask was created from the regions of the image that satisfied both criteria. The mask area was converted from pixels into mm.

### Statistical Analyses

Statistical analyses were performed using either R v4.0.0 (Team, 2020) or GraphPad Prism v8 (GraphPad Software, Inc.). Statistical significance was set at *p* ≤ 0.05. For the short-term study, comparisons of flux between PBS and LPC groups were performed using Welch two sample t-tests on log-transformed data. To compare flux between the PBS and LPC treatments for the long-term study, a hurdle regression model was fitted using the *hurdle* function in the *pscl* package in R (Zeileis et al., 2008). The hurdle regression model is a two-part model; a binary logit model to distinguish between the positive counts and those below the detection limit (assigned the value 100), and a zero-truncated negative binomial model for the positive counts. Treatment (PBS, LPC) was fitted as the regressor in both parts of the model. Differences between the groups were assessed using a Wald Chi-Squared test. The LacZ staining area measurements from the *en face* images was analysed using a one-way ANOVA with Tukey’s multiple comparisons test.

## Results

### Animal Procedures

Chemical and physical airway perturbation techniques were well-tolerated by the rats with all animals surviving the procedures. Animal weight, health and wellbeing characteristics were assessed daily for the duration of the study using a score-based animal monitoring system, with no significant effects on animal health noted. Expected transient weight loss was similar in all experimental groups (LPC, physical and LV-only), irrespective of whether airway perturbation was performed (data not shown).

### Luciferase Gene Expression Assessed 1-week Post Gene Transfer

**FIGURE 2:**
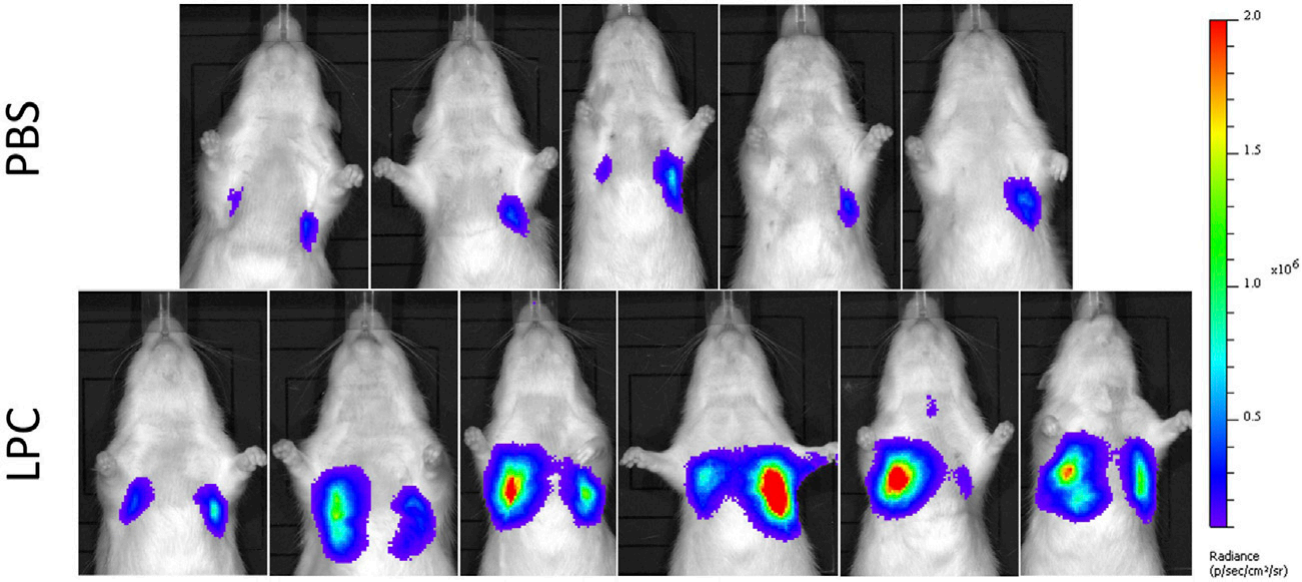
*In vivo* bioluminescence images acquired 1-week following LV-FLAG-Luc-GFP delivery in PBS-sham and LPC conditioned rats. Localisation of bioluminescence *in vivo* was predominantly in the lung region in both PBS and LPC pre-treated animals.

**FIGURE 3:**
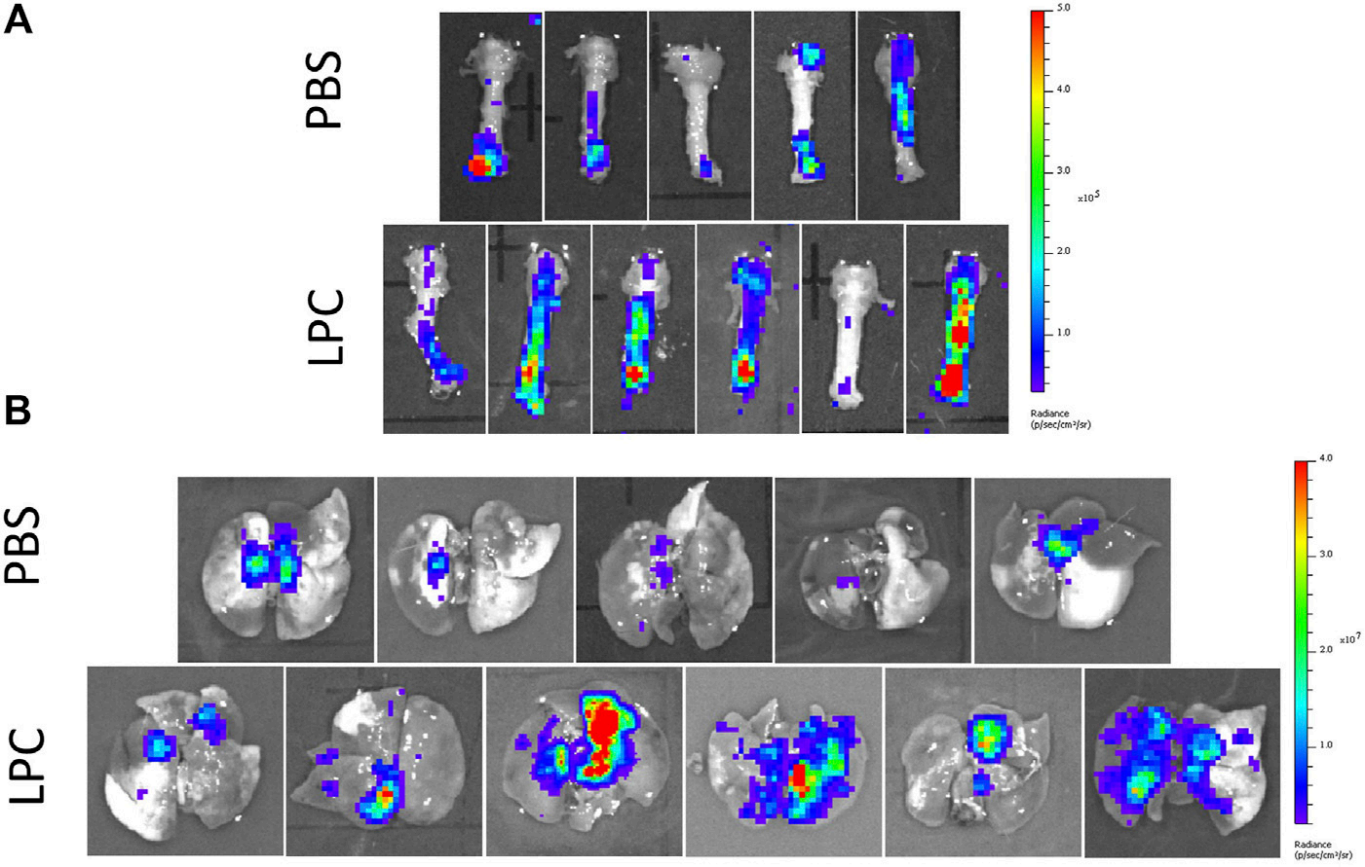
*Ex vivo* bioluminescence images acquired 1-week following LV-FLAG-Luc-GFP delivery in PBS-sham and LPC conditioned rats. *Ex vivo* images of **(A)** the trachea and **(B)** lungs following removal of the trachea.

**FIGURE 4:**
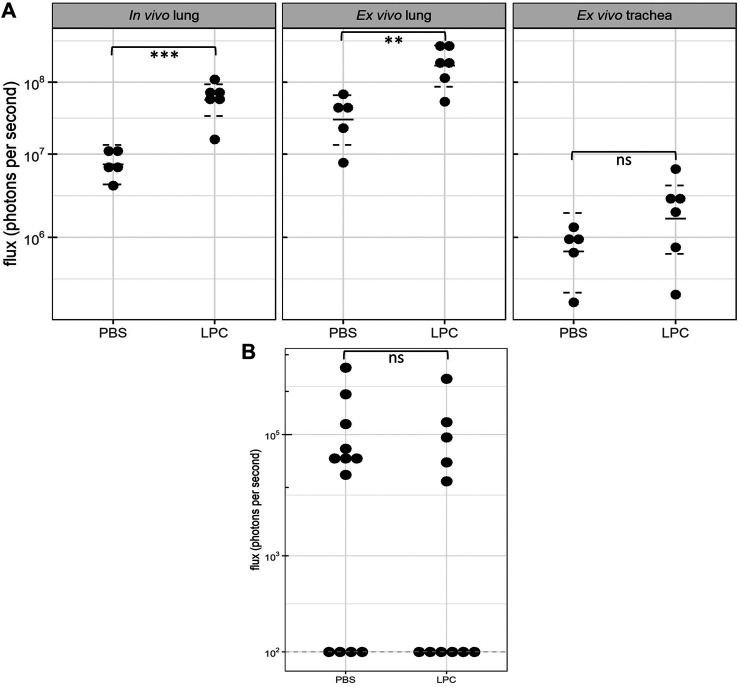
Bioluminescence flux (log scale) acquired 1-week and 12 months following LV-FLAG-Luc-GFP delivery in PBS-sham and LPC conditioned rats. **(A)** *In vivo* and *ex vivo* bioluminescence flux acquired 1-week post gene transfer. Statistical analyses of the *in vivo and ex vivo* lung data revealed significant differences in flux between PBS and LPC conditioned groups, with *p* = 0.0002 and *p* = 0.005, respectively. Statistical analysis of the *ex vivo* trachea indicated there was no significant difference in flux between LPC and PBS groups, *p* = 0.2. The plot shows the estimated median and 95% confidence intervals. *n* = 5-6 animals per group, Welch two sample *t*-test, ns = not significant, **p ≤ 0.01, ***p ≤ 0.001. **(B)** *Ex vivo* lung bioluminescence flux acquired 12 months following gene transfer. The dashed line indicates the limit of detection of the IVIS machine (10^2^). No significant difference in flux was observed between animals in the PBS and LPC groups*, p* = 0.6. *n* = 11–12 animals per group, Wald Chi-squared test, ns = not significant.

In the short-term assessment, rats received either PBS or LPC conditioning and were imaged 1-week following a single LV gene delivery. Bioluminescence was predominantly observed in the lungs *in vivo*, with only one animal exhibiting signal in the trachea region (Figure 2). Statistical analysis of the *in vivo* data revealed that LPC conditioning resulted in significantly higher flux when compared to animals that received the PBS sham treatment (Figure 4A). Upon excision of the tissues and subsequent imaging, bioluminescence was observed in the trachea (Figure 3A) and lungs (Figure 3B) of all animals. Analysis of the *ex vivo* data indicated that there was no difference in flux between the LPC and PBS conditioned groups for the trachea, while the excised lungs revealed that LPC airway conditioned rats had significantly greater flux levels (Figure 4A), regardless of the sex of the animals.

### Luciferase Gene Expression Assessed 12-months Post Gene Transfer

In the long-term study assessing chemical conditioning, few animals from either the LPC or PBS group had detectable levels of *in vivo* bioluminescence at the 12-month time point (data not shown), therefore statistical comparisons could not be performed on this data. *Ex vivo* imaging of the trachea also revealed insufficient animals with bioluminescence present to perform statistical analysis. Imaging of the *ex vivo* lungs showed that close to half the animals from both LPC and PBS groups had values recorded at the limit of bioluminescence detection. Additionally, the flux levels measured in the *ex vivo* lungs at 12 months were substantially lower compared to the animals imaged at 1-week post-delivery. *Ex vivo* lung imaging revealed that there was no significant difference in flux between LPC conditioned and PBS-sham animals’ 12-months following LV-delivery (Figure 4B).

### Tracheal LacZ Gene Expression Following Chemical Conditioning and Physical Perturbation

**FIGURE 5:**
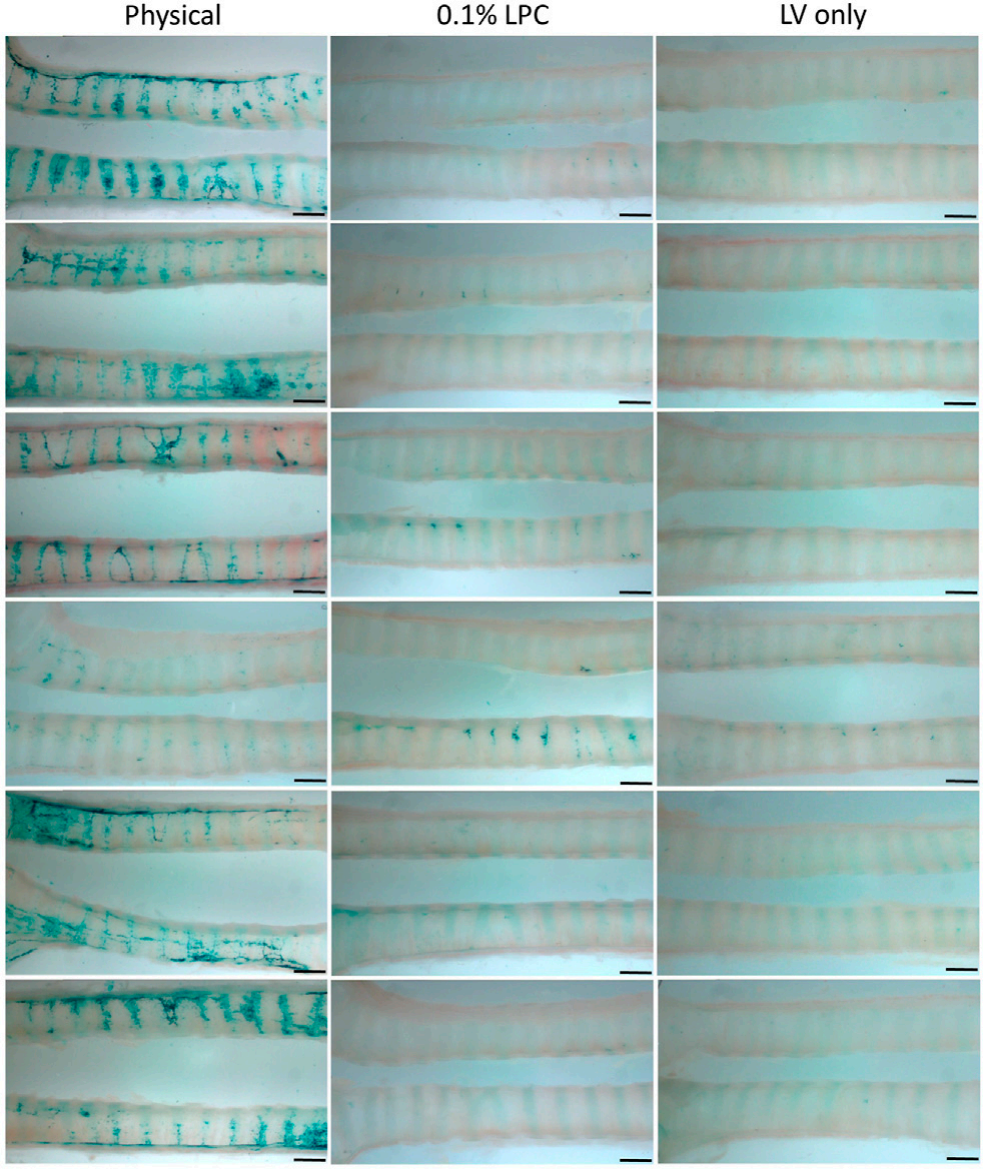
Comparison of *en face* LacZ staining using physical perturbation, chemical conditioning with 0.1% LPC, or LV vector-only assessed 1-week post gene transfer. Images show the trachea from each individual animal longitudinally cut into two sections to reveal the LacZ staining present within the lumen. The images display the middle portion of the trachea and are oriented to show the proximal trachea on the right. *n =* 6 rats per group. Scale bar = 1.5 mm.

**FIGURE 6:**
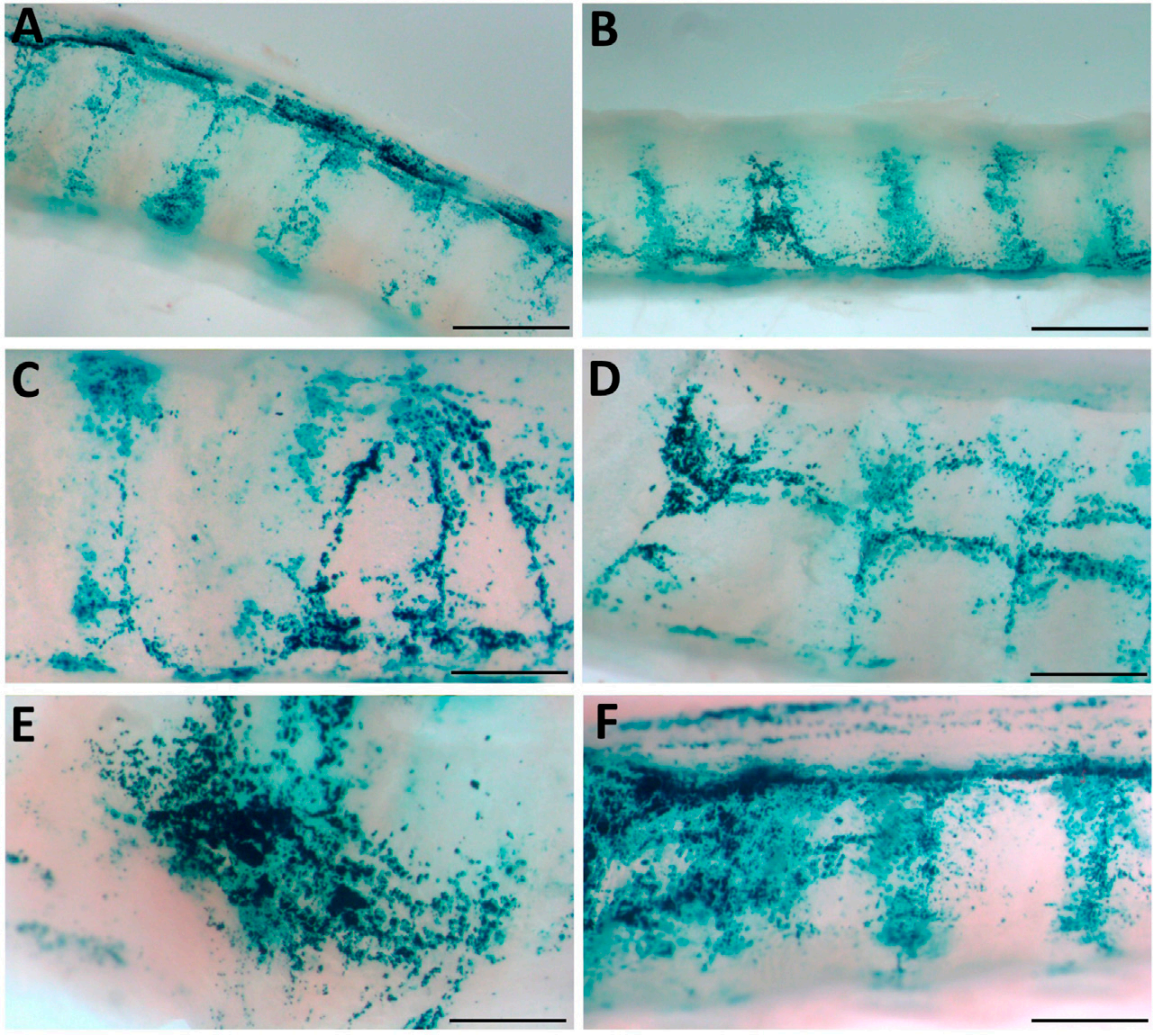
Physical perturbation reveals varying patterns of LacZ staining. **(A, B)** LacZ staining is increased in the areas overlying the inter-cartilage segments **(C**–**F)** Basket-induced perturbation creates regions of strong staining. High magnification images were acquired from the samples shown in Figure 5 and indicate staining patterns present in multiple animals. Scale bars A, B = 1.5 mm; C-F = 0.75 mm.

*En face* observations of the trachea revealed different levels of LacZ staining as a result of the perturbation or conditioning method used. Visually, rats that received physical perturbation prior to LV-delivery demonstrated greatly enhanced LacZ staining in the trachea when compared to rats that received LPC conditioning or LV vector-only (Figure 5). Interestingly, one animal in the physical perturbation group exhibited much lower staining levels compared to the others, indicative of potential variability with this technique. The transduction patterns observed as a result of physical perturbation were not uniform in appearance. Striated regions of increased staining over the intercartilaginous ligaments were apparent in all specimens, possibly reflecting the presence of cilia-rich zones that overlay the ligament segments in rats (Oliveira et al., 2003) and preferential transduction of ciliated cells by the VSV-G pseudotyped LV gene transfer vector. Varying LacZ staining patterns and strong expression were observed in animals that received physical perturbation (Figures 6A–F). Notably, rats in the LPC and LV-only groups demonstrated the highest levels of staining in the proximal trachea (Supplementary Figure S1), likely due to unintentional ET tube-induced epithelial damage in this region, providing further support for the effectiveness of physical perturbation in boosting airway gene transfer.

**FIGURE 7:**
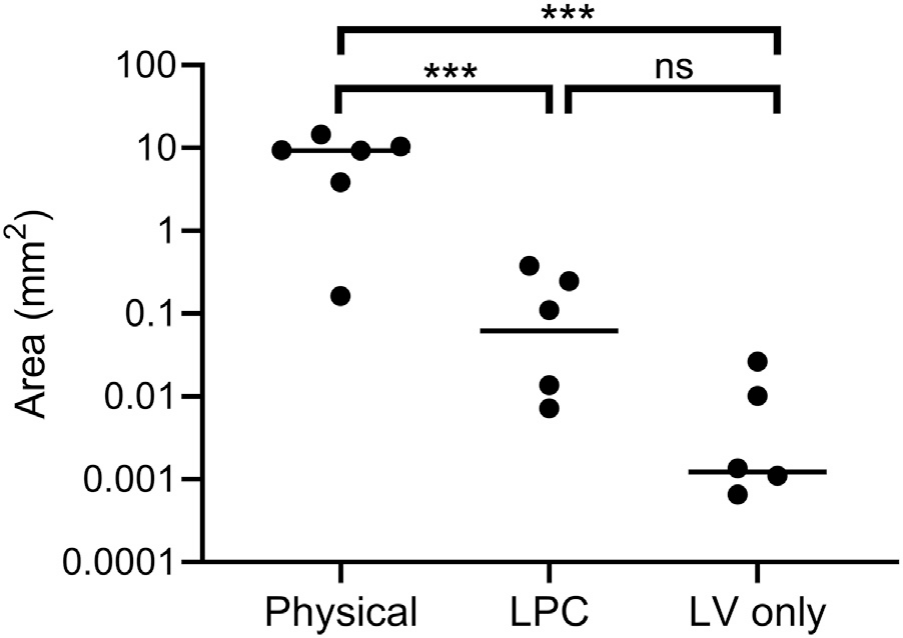
Quantification of LacZ staining area (log scale) from *en face* trachea images shown in Figure 5. Physical perturbation had a significantly greater area of tracheal LacZ staining after 1-week when compared to LPC conditioned (*p =* 0.001) and LV vector-only control animals (*p =* 0.0008). There was no significant difference in the area of LacZ staining between LPC conditioned animals and those that received only LV vector (*p =* 0.1). The plot indicates the median. *n* = 6 animals per group, one-way ANOVA with Tukey’s post-hoc test, ns = not significant, ***p ≤ 0.001. Note that two animals are not observable on the plot as the quantified area was zero.

Digital quantification of the blue-green coloured regions in the *en face* trachea images demonstrated significantly greater levels of LacZ staining in the physical perturbation group, when compared to LPC conditioned and LV vector-only control rats (Figure 7). The physical damage group demonstrated more than a 1000-fold increase in the area of LacZ staining when compared to the control group. There was no statistically significant difference in staining area between LPC conditioned rats and controls that received only LV vector with no epithelial disruption prior to delivery.

### Histological Observations Arising From Physical Perturbation

**FIGURE 8:**
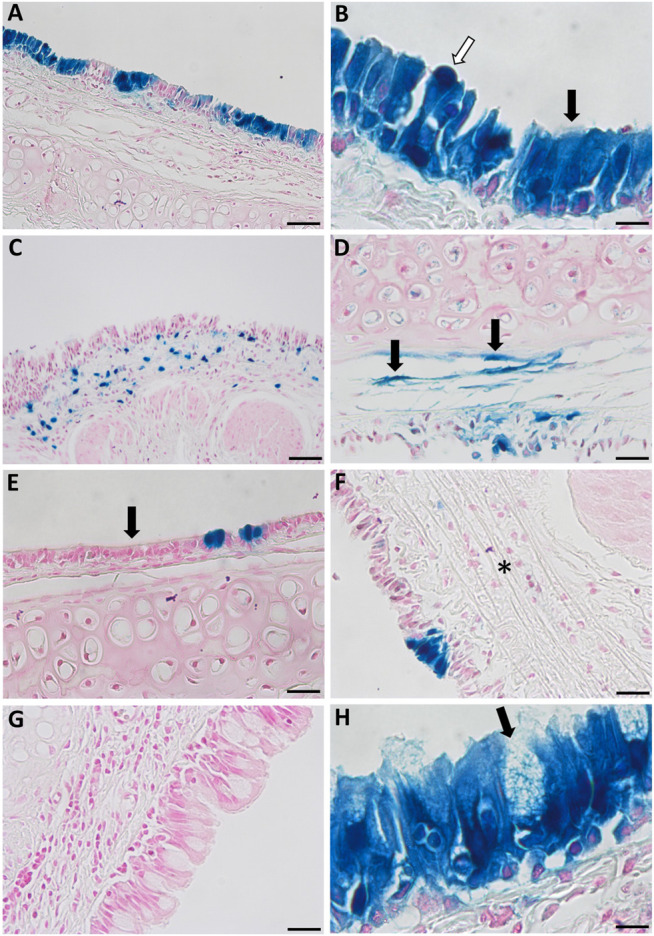
Histological observations in the tracheal epithelium of rats that received physical perturbation prior to LV-LacZ vector delivery assessed 1-week post gene transfer. **(A)** LacZ staining was discontinuous throughout the tracheal epithelium with some areas of intense staining observed. **(B)** A range of LacZ-positive surface epithelial cell types were noted including ciliated (black arrow) and non-ciliated (white arrow) cells. Transduced regions were noted within the lamina propria including **(C)** suspected macrophages and **(D)** connective tissue. While most of the tissue demonstrated full regeneration, some regions showed **(E)** a flattened epithelial layer lacking pseudostratification, while other regions demonstrated **(F)** connective tissue proliferation (fibrosis) in response to healing (denoted by asterisk). **(G)** Goblet cell hyperplasia was found at the carina of two animals with **(H)** a proportion of these goblet cells exhibiting LacZ staining. Goblet cells were identified by a distinct goblet shape and granules filled with mucus. Images are shown from selected animals. Nuclear fast red counterstain. Scale bar = 10 μM.

**FIGURE 9:**
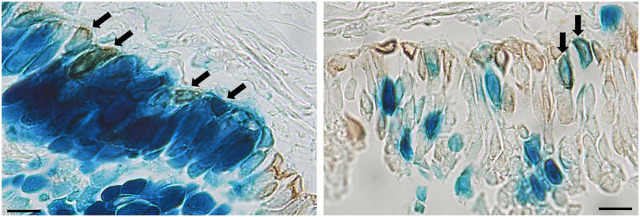
Immunohistochemical detection of basal cells in rats that received physical perturbation prior to LV-LacZ vector delivery assessed 1-week post gene transfer. Basal cells were identified by immunohistochemical staining with anti-cytokeratin 5 antibody as shown by brown staining. Examples of transduced basal cells are indicated by arrows. Scale bar = 10 μM.

A range of different cell types were transduced as a result of performing physical perturbation prior to LV vector delivery. LacZ-positive ciliated and non-ciliated cells were observed in the tracheal epithelium (Figures 8A,B) and immunohistochemical staining for cytokeratin 5 confirmed the presence of transduced basal cells (Figure 9). Cells within the lamina propria were also transduced, including suspected macrophages (Figure 8C) and regions of connective tissue (Figure 8D). Histologically, the tracheal tissue demonstrated evidence of repair processes caused by damage to the epithelium and surrounding areas. Most regions of the tracheal epithelium had fully regenerated by 1-week post treatment, consistent with the timing observed in previous studies (Shimizu et al., 1994; Pickles et al., 1996). However, occasionally areas of epithelium were found to still be undergoing repair, as indicated by an attenuated appearance, lack of cilia and rounding of the columnar cells (Figure 8E), or in other regions, proliferation of connective tissue (Figure 8F). Goblet cell hyperplasia was also apparent at the carina and upper bronchi of two animals (Figure 8G). Interestingly, many of these hyperplastic goblet cells appeared to be LacZ-positive (Figure 8H).

## Discussion

Gene-addition therapy is a promising option for treatment of certain pulmonary diseases, but there are still many challenges to successful translation to the clinic. Adequate delivery of therapeutic agents to cells of the airway epithelium remains one of the greatest challenges to achieving effective lung-based genetic therapies, including gene-addition and gene editing strategies. The lung has evolved mechanisms to resist invasion by foreign bodies, with the airway epithelium acting as a physical barrier that significantly reduces the efficacy of airway gene transfer. Many viral vectors used for airway gene therapies have a natural tropism toward receptors located on the basolateral membrane and thus produce limited transduction *via* the apical surface when applied to an intact airway epithelium.

To overcome inefficient transduction, strategies have been employed to disrupt the integrity of the airway epithelium prior to gene delivery, allowing the vector access to basolateral membrane-located receptors, and to cells that are not in direct contact with the airway lumen such as basal cells (Pickles, 2004). Given our recent generation of two cystic fibrosis rat models (McCarron et al., 2020), the work reported here employed normal rats to develop and assess chemical and physical airway perturbation methods. In these studies, the trachea was used a surrogate for the lower conducting airways, as it is easily accessible for physical perturbation techniques and the cellular architecture is similar to human lower airways (Mercer et al., 1994).

In this work, the effectiveness of LPC conditioning was assessed for the first time in rat airways. While mouse studies have shown enhanced gene expression when using LPC conditioning prior to LV gene transfer (Cmielewski et al., 2014; Cmielewski et al., 2017), its effects in rat airways were unknown. In this study, gene expression levels were found to be significantly increased in the lungs of rats that received LPC conditioning when assessed 1-week post vector delivery. Upon *in vivo* imaging, only one animal exhibited bioluminescence signal within the trachea, but following excision of the tissues and subsequent *ex vivo* imaging, bioluminescence was observed in the trachea of all animals, albeit at varying levels. When imaged *ex vivo* the flux emitted from the trachea was substantially lower than the *ex vivo* lung, therefore the low bioluminescence in combination with the need to detect the signal through the overlying skin and tissue may explain the lack of tracheal bioluminescence upon *in vivo* imaging. Notably, when the trachea was excised and imaged, LPC did not appear to enhance bioluminescence flux when compared to the PBS-sham group.

Long-term gene expression was assessed in a separate cohort of rats, which revealed that *luciferase* gene expression persisted in the lungs of some animals for up to 12 months following a single LV-delivery, irrespective of whether LPC airway conditioning was performed. These two studies indicate that while LPC conditioning initially improves transduction levels in the lungs, it does not increase the longevity of gene expression, suggesting that basal cell transduction was not substantially enhanced with use of LPC. Nevertheless, it is likely that basal cells, or other known lung stem cell types (e.g., club cells, alveolar type II cells), were transduced in animals from both LPC and PBS-sham groups, as the duration of gene expression exceeds the four month lifespan of terminally differentiated tracheal-bronchial epithelium described in adult rodents (Rawlins and Hogan, 2006).

While LV-mediated gene transfer demonstrated persistent *luciferase* gene expression in a proportion of rats, many individuals from both the LPC conditioning and PBS-sham groups had undetectable flux by 12 months. The lack of gene expression observed at the 12 month time point could potentially be attributed to transgene silencing (Ellis, 2005), alternatively, loss of gene-expressing cells with natural cell-turnover may explain this observation. We do not have data that can explain why some individuals responded better than others, though this phenomenon is consistently observed in airway gene therapy animal studies. Possible factors include but are not limited to, the proportion of basal cells transduced, varying host immune responses to the vector and formulation, size of the animal (which dictates the surface area to vector volume ratio), vector-fluid dynamics and animal respiration rate/depth, which can result in variable gene vector distribution within the airways, and the amount of time that the vector is in contact with the airway surface. An improved understanding of the basis of these variable individual responses to airway gene-addition therapy would provide significant value for progressing to clinical trials.

In the second study employing LV-LacZ vector, physical perturbation of the airway epithelium resulted in very high levels of gene transduction in the trachea when compared to animals receiving LPC chemical conditioning or LV vector-only. Moreover, LPC conditioning did not significantly enhance tracheal LacZ expression above the control (LV-only) group, consistent with the short-term bioluminescence study where LPC use did not result in a significant increase in tracheal flux when compared with the PBS-sham group. However, it is important to note that the LPC conditioning protocol used here had been previously established for mice, therefore the concentration or volume employed may not have been optimal for rats.

As with many viral receptors, the VSV-G receptor (low-density lipoprotein) is located on the basolateral surface, and transduction *via* the apical surface is typically low level (Finkelshtein et al., 2013). While the mechanisms here are not fully understood, it is likely that physical perturbation increases gene transfer *via* two potential routes: 1) disruption of the integrity of the epithelial tight-junctions allowing the gene transfer vector entry to epithelial cells *via* basolateral receptors, or 2) removal of transduction-resistant columnar cells and exposure of more susceptible cell types on the basement membrane (e.g., basal cells). In this study, physical damage was confirmed to produce basal cell transduction. Additionally, both *en face* and histological examination revealed distinct clusters of LacZ-positive cells, reminiscent of basal cell clonal expansion observed in previous studies of induced regeneration following polidocanol epithelial stripping (Mitomo et al., 2010; Farrow et al., 2018). These clusters suggest that one or more basal stem cells have been transduced and subsequently differentiated into a LacZ expressing pseudostratified epithelium, consistent with previous studies showing the timing of epithelial regeneration in rat airways following mechanical injury (Shimizu et al., 1994). However, further work including lineage tracing studies would be necessary to confirm this hypothesis. Alternatively, it is possible that clusters of LacZ-expressing cells may be due to strong focal damage or repetitive contact with the basket, making these areas more susceptible to LV-transduction.

Physical perturbation of the airway surface may provide gene transfer vectors with improved access to basal cells. An airway gene-addition therapy that transduces only the terminally differentiated airway surface cells could result in an inevitable waning of transgene expression with normal cell turnover, and the need for vector readministration. While repeated administration is unlikely to be completely avoided, multiple deliveries of a viral-based gene therapy is undesirable as immune responses may be elicited thus reducing the efficacy of subsequent doses (Sinn et al., 2009; Mitomo et al., 2010). Basal cells are multipotent stem cells of the conducting airways and have the capacity for self-renewal, clonal expansion, and the ability to differentiate into epithelial cells types to maintain homeostasis and repair following injury (Hong et al., 2004; Rock et al., 2009; Rock et al., 2010; Hogan et al., 2014). Successful transduction of basal cells has the ability to produce enduring gene expression, as the gene-corrected progeny will repopulate the surface epithelium following natural cell turnover. It is therefore desirable to develop an airway gene-addition therapy that not only produces efficient gene transfer but can also transduce basal cells (Cao et al., 2018; Farrow et al., 2018).

Following use of physical perturbation, cells within some regions of the tracheal lamina propria appeared to be transduced. While a certain degree of epithelial injury was expected, these findings suggest that in some areas the physical perturbation extended too far into the airway surface, likely due to forces produced by the wire basket or repetitive contact. Goblet cell hyperplasia was also noted in some animals, a protective response that can occur as a result of mucosal surface irritation (Herbert et al., 2018). Notably, the animals that displayed goblet cell hyperplasia had many LacZ-positive goblet cells within these regions, suggesting that intact basal cells were transduced and subsequently differentiated into goblet cells upon epithelial regeneration. Our previous short-term assessments have shown that VSV-G pseudotyped LV vectors rarely transduce goblet cells (Limberis et al., 2002; Farrow et al., 2018), so their presence further indicates likely expansion and differentiation of transduced basal cells.

One major benefit of physical perturbation noted in this study is the ability to achieve high transduction levels from a relatively small number of LV particles per animal (1 × 10^8^ TU). By using physical epithelial disruption methods to improve gene transfer efficacy, the LV titres required to achieve therapeutic levels of gene correction could be reduced, thus improving the feasibility of an *in vivo* gene therapy approach. Moreover, such reductions in vector titre would provide the benefits of lower LV production time and cost, as well as lessened immune responses. The advantages of physical perturbation could also be applied to improving the efficacy of other lung-based genetic therapies including delivery of gene editing machinery, nanoparticle-based gene transfer systems, and stem cell-transplantation strategies. For instance, airway engraftment of transplanted cells could be facilitated *via* use of physical perturbation methods rather than chemical agents, which have previously been employed pre-clinically to achieve epithelial disruption (King et al., 2020), but have disadvantages such as toxicity and poor delivery control.

While the results of this tracheal physical perturbation study provide promising proof-of-principle data, the target for cystic fibrosis airway gene-addition therapy will be the lower conducting airways, as this is where disease pathophysiology occurs. Accordingly, further pre-clinical work in rats is needed to assess the feasibility of this approach in the lower airways, with our previously established rat bronchoscopy methods offering a potential option for enabling access to these small airways for perturbation. Our minimally invasive miniature bronchoscope technique provides access to at least the fourth generation of branching in an adult rat, therefore perturbation-devices could be guided *via* the bronchoscope working channel to the small airways (McIntyre et al., 2018). In addition to rat studies, investigations using larger animals such as pigs or sheep will be necessary to identify the features of a perturbation device that would be considered safe and effective for human clinical use.

Histological assessment of the airway tissue immediately post-damage, and the time-course of epithelial regeneration following physical perturbation, will also provide value for understanding the mechanisms that result in improved gene transfer with this approach. Future studies should aim to establish more refined techniques to improve control and reduce the intensity of the perturbation, so that off-target cell transduction and disruption to the subepithelial tissue is minimised. Ultimately, a careful balance between safety, tolerability, and efficacy aspects will be critical in pursuing this type of physical perturbation approach for human airway gene therapy clinical trials. In the interim however, there remains considerable research value in developing physical perturbation approaches for pre-clinical investigation.

In summary, although LPC conditioning initially increased gene expression levels in rat lungs, it did not appear to improve reporter gene expression longevity. Even without conditioning, a proportion of rats had measurable flux present in the lungs 12 months following dosing, indicating that a single LV delivery can provide persistent gene expression, albeit at low levels. Physical perturbation to the trachea immediately prior to LV-mediated gene transfer resulted in a 1000-fold increase in LacZ staining when compared to animals that did not receive any airway perturbation, demonstrating that this approach can provide significant benefit for improving gene transfer efficacy and warrants further development and investigation.

## Data Availability

The raw data supporting the conclusions of this article will be made available by the authors, without undue reservation.

## References

[B1] Anson D. S. McIntyre C. Thomas B. Koldej R. Ranieri E. Roberts A. (2007). Lentiviral-mediated Gene Correction of Mucopolysaccharidosis Type IIIA. Genet. Vaccin. Ther. 5, 1. 10.1186/1479-0556-5-1 PMC1783652 17227588

[B2] Bandeira V. Peixoto C. Rodrigues A. F. Cruz P. E. Alves P. M. Coroadinha A. S. (2012). Downstream Processing of Lentiviral Vectors: Releasing Bottlenecks. Hum. Gene Ther. Methods 23, 255–263. 10.1089/hgtb.2012.059 22934827

[B3] Cao H. Machuca T. N. Yeung J. C. Wu J. Du K. Duan C. (2013). Efficient Gene Delivery to Pig Airway Epithelia and Submucosal Glands Using Helper-dependent Adenoviral Vectors. Mol. Ther. - Nucleic Acids 2, e127. 10.1038/mtna.2013.55 24104599 PMC3890457

[B4] Cao H. Ouyang H. Grasemann H. Bartlett C. Du K. Duan R. (2018). Transducing Airway Basal Cells with a Helper-dependent Adenoviral Vector for Lung Gene Therapy. Hum. Gene Ther. 29, 643–652. 10.1089/hum.2017.201 29320887

[B5] Castellani S. Conese M. (2010). Lentiviral Vectors and Cystic Fibrosis Gene Therapy. Viruses 2, 395–412. 10.3390/v2020395 21994643 PMC3185599

[B6] Chu Q. St. George J. A. Lukason M. Cheng S. H. Scheule R. K. Eastman S. J. (2001). EGTA Enhancement of Adenovirus-Mediated Gene Transfer to Mouse Tracheal Epitheliumin Vivo. Hum. Gene Ther. 12, 455–467. 10.1089/104303401300042348 11268280

[B7] Cmielewski P. Anson D. S. Parsons D. W. (2010). Lysophosphatidylcholine as an Adjuvant for Lentiviral Vector Mediated Gene Transfer to Airway Epithelium: Effect of Acyl Chain Length. Respir. Res. 11, 84. 10.1186/1465-9921-11-84 20569421 PMC2905357

[B8] Cmielewski P. Donnelley M. Parsons D. W. (2014). Long-term Therapeutic and Reporter Gene Expression in Lentiviral Vector Treated Cystic Fibrosis Mice. J. Gene Med. 16, 291–299. 10.1002/jgm.2778 25130650

[B9] Cmielewski P. Farrow N. Devereux S. Parsons D. Donnelley M. (2017). Gene Therapy for Cystic Fibrosis: Improved Delivery Techniques and Conditioning with Lysophosphatidylcholine Enhance Lentiviral Gene Transfer in Mouse Lung Airways. Exp. Lung Res. 43, 426–433. 10.1080/01902148.2017.1395931 29236544

[B10] Copreni E. Penzo M. Carrabino S. Conese M. (2004). Lentivirus-mediated Gene Transfer to the Respiratory Epithelium: a Promising Approach to Gene Therapy of Cystic Fibrosis. Gene Ther. 11, 67–75. 10.1038/sj.gt.3302372 15454960

[B11] Ellis J. (2005). Silencing and Variegation of Gammaretrovirus and Lentivirus Vectors. Hum. Gene Ther. 16, 1241–1246. 10.1089/hum.2005.16.1241 16259557

[B12] Farrow N. Donnelley M. Cmielewski P. Roscioli E. Rout-Pitt N. McIntyre C. (2018). Role of Basal Cells in Producing Persistent Lentivirus-Mediated Airway Gene Expression. Hum. Gene Ther. 29, 653–662. 10.1089/hum.2017.059 29179571

[B13] Finkelshtein D. Werman A. Novick D. Barak S. Rubinstein M. (2013). LDL Receptor and its Family Members Serve as the Cellular Receptors for Vesicular Stomatitis Virus. Proc. Natl. Acad. Sci. 110, 7306–7311. 10.1073/pnas.1214441110 23589850 PMC3645523

[B14] Flotte T. R. Ng P. Dylla D. E. McCray P. B. Jr. Wang G. Kolls J. K. (2007). Viral Vector-Mediated and Cell-Based Therapies for Treatment of Cystic Fibrosis. Mol. Ther. 15, 229–241. 10.1038/sj.mt.6300002 17235299

[B15] Gregory L. G. Harbottle R. P. Lawrence L. Knapton H. J. Themis M. Coutelle C. (2003). Enhancement of Adenovirus-Mediated Gene Transfer to the Airways by DEAE Dextran and Sodium Caprate In Vivo. Mol. Ther. 7, 19–26. 10.1016/s1525-0016(02)00021-7 12573614

[B16] Herbert R. A. Janardhan K. S. Pandiri A. R. Cesta M. F. Miller R. A. (2018). “Chapter 22 - Nose, Larynx, and Trachea,” in Boorman's Pathology of the Rat. Second Edition. Editor Suttie AW. (Boston: Academic Press), 391–435.

[B17] Hogan B. L. M. Barkauskas C. E. Chapman H. A. Epstein J. A. Jain R. Hsia C. C. W. (2014). Repair and Regeneration of the Respiratory System: Complexity, Plasticity, and Mechanisms of Lung Stem Cell Function. Cell Stem Cell 15, 123–138. 10.1016/j.stem.2014.07.012 25105578 PMC4212493

[B18] Hong K. U. Reynolds S. D. Watkins S. Fuchs E. Stripp B. R. (2004). Basal Cells Are a Multipotent Progenitor Capable of Renewing the Bronchial Epithelium. Am. J. Pathol. 164, 577–588. 10.1016/S0002-9440(10)63147-1 14742263 PMC1602270

[B19] Johnson L. G. Olsen J. C. Naldini L. Boucher R. C. (2000). Pseudotyped Human Lentiviral Vector-Mediated Gene Transfer to Airway Epithelia In Vivo. Gene Ther. 7, 568–574. 10.1038/sj.gt.3301138 10819571

[B20] Kim N. Duncan G. A. Hanes J. Suk J. S. (2016). Barriers to Inhaled Gene Therapy of Obstructive Lung Diseases: A Review. J. Controlled Release 240, 465–488. 10.1016/j.jconrel.2016.05.031 PMC5064827 27196742

[B21] King N. E. Suzuki S. Barillà C. Hawkins F. J. Randell S. H. Reynolds S. D. (2020). Correction of Airway Stem Cells: Genome Editing Approaches for the Treatment of Cystic Fibrosis. Hum. Gene Ther. 31, 956–972. 10.1089/hum.2020.160 32741223 PMC7495916

[B22] Koehler D. R. Frndova H. Leung K. Louca E. Palmer D. Ng P. (2005). Aerosol Delivery of an Enhanced Helper-dependent Adenovirus Formulation to Rabbit Lung Using an Intratracheal Catheter. J. Gene Med. 7, 1409–1420. 10.1002/jgm.797 15999396

[B23] Li J. Bonneau L. Zimmerman J. Weiss D. (2007). Perfluorochemical (PFC) Liquid Enhances Recombinant Adenovirus Vector-Mediated Viral Interleukin-10 (AdvIL-10) Expression in Rodent Lung. J. Inflamm. 4, 9. 10.1186/1476-9255-4-9 PMC1868755 17472748

[B24] Limberis M. Anson D. S. Fuller M. Parsons D. W. (2002). Recovery of Airway Cystic Fibrosis Transmembrane Conductance Regulator Function in Mice with Cystic Fibrosis after Single-Dose Lentivirus-Mediated Gene Transfer. Hum. Gene Ther. 13, 1961–1970. 10.1089/10430340260355365 12427306

[B25] McCarron A. Cmielewski P. Reyne N. McIntyre C. Finnie J. Craig F. (2020). Phenotypic Characterization and Comparison of Cystic Fibrosis Rat Models Generated Using CRISPR/Cas9 Gene Editing. Am. J. Pathol. 190, 977–993. 10.1016/j.ajpath.2020.01.009 32084371

[B26] McCarron A. Donnelley M. McIntyre C. Parsons D. (2019). Transient Lentiviral Vector Production Using a Packed-Bed Bioreactor System. Hum. Gene Ther. Methods 30, 93–101. 10.1089/hgtb.2019.038 31084376

[B27] McIntyre C. Donnelley M. Rout-Pitt N. Parsons D. (2018). Lobe-Specific Gene Vector Delivery to Rat Lungs Using a Miniature Bronchoscope. Hum. Gene Ther. Methods 29, 228–235. 10.1089/hgtb.2018.050 29993287

[B28] Mercer R. R. Russell M. L. Roggli V. L. Crapo J. D. (1994). Cell Number and Distribution in Human and Rat Airways. Am. J. Respir. Cell Mol. Biol. 10, 613. 10.1165/ajrcmb.10.6.8003339 8003339

[B29] Mitomo K. Griesenbach U. Inoue M. Somerton L. Meng C. Akiba E. (2010). Toward Gene Therapy for Cystic Fibrosis Using a Lentivirus Pseudotyped with Sendai Virus Envelopes. Mol. Ther. 18, 1173–1182. 10.1038/Mt.2010.13 20332767 PMC2889732

[B30] Oliveira M. J. R. Pereira A. S. Guimarães L. Grande N. R. Moreira de Sá C. Aguas A. P. (2003). Zonation of Ciliated Cells on the Epithelium of the Rat Trachea. Lung 181, 275–282. 10.1007/s00408-003-1030-1 14705771

[B31] Pickles R. J. (2004). Physical and Biological Barriers to Viral Vector-Mediated Delivery of Genes to the Airway Epithelium. Proc. Am. Thorac. Soc. 1, 302–308. 10.1513/pats.200403-024MS 16113450

[B32] Pickles R. J. Barker P. M. Ye H. Boucher R. C. (1996). Efficient Adenovirus-Mediated Gene Transfer to Basal but Not Columnar Cells of Cartilaginous Airway Epithelia. Hum. Gene Ther. 7, 921–931. 10.1089/hum.1996.7.8-921 8727506

[B33] Rawlins E. L. Hogan B. L. (2006). Epithelial Stem Cells of the Lung: Privileged Few or Opportunities for Many? Development 133, 2455–2465. 10.1242/dev.02407 16735479

[B34] Rock J. R. Onaitis M. W. Rawlins E. L. Lu Y. Clark C. P. Xue Y. (2009). Basal Cells as Stem Cells of the Mouse Trachea and Human Airway Epithelium. Proc. Natl. Acad. Sci. 106, 12771. 10.1073/pnas.0906850106 19625615 PMC2714281

[B35] Rock J. R. Randell S. H. Hogan B. L. M. (2010). Airway Basal Stem Cells: a Perspective on Their Roles in Epithelial Homeostasis and Remodeling. Dis. Models Mech. 3, 545–556. 10.1242/dmm.006031 PMC2931533 20699479

[B36] Rout-Pitt N. McCarron A. McIntyre C. Parsons D. Donnelley M. (2018). Large-scale Production of Lentiviral Vectors Using Multilayer Cell Factories. J. Biol. Methods 5, e90. 10.14440/jbm.2018.236 31453241 PMC6706103

[B37] Shimizu T. Nishihara M. Kawaguchi S. Sakakura Y. (1994). Expression of Phenotypic Markers during Regeneration of Rat Tracheal Epithelium Following Mechanical Injury. Am. J. Respir. Cell Mol Biol 11, 85–94. 10.1165/ajrcmb.11.1.7517145 7517145

[B38] Sinn P. L. Burnight E. R. McCray P. B. Jr. (2009). Progress and Prospects: Prospects of Repeated Pulmonary Administration of Viral Vectors. Gene Ther. 16, 1059–1065. 10.1038/gt.2009.87 19641533 PMC4376355

[B39] Team R. C. (2020). A Language and Environment for Statistical Computing. Vienna, Austria: R Foundation for Statistical Computing. *2012* [Online]. Available at: https://www.r-project.org/ (Accessed November 20th, 2020).

[B40] Zeileis A. Kleiber C. Jackman S. (2008). Regression Models for Count Data in R. J. Stat. Softw. 27, 1–25. 10.18637/jss.v027.i08

