# Supplementary material for: Breaching the Delivery Barrier: Chemical and Physical Airway Epithelium Disruption Strategies for Enhancing Lentiviral-Mediated Gene Therapy
Source: Front Pharmacol. 2021 Apr 26;12:669635. doi: 10.3389/fphar.2021.669635 (PMC8107471; doi:10.3389/fphar.2021.669635)
Supplement: Supplementary file 1 [file Image1.pdf]

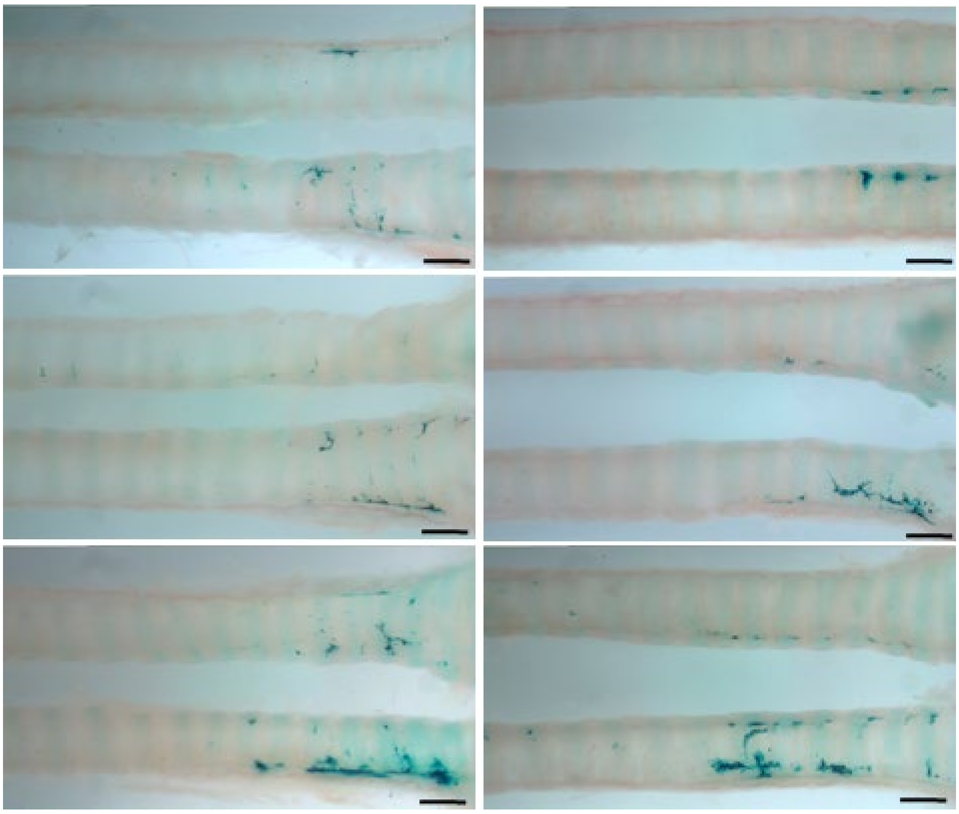

Supplementary Figure 1: *En face* images from animals receiving 0.1% LPC conditioning and LV vector-only indicate strong regions of LacZ staining in the proximal trachea at the site of unintentional ET tube damage.
